# Antimicrobial peptide DP7 with potential activity against SARS coronavirus infections

**DOI:** 10.1038/s41392-021-00551-1

**Published:** 2021-04-01

**Authors:** Rui Zhang, Xiaohua Jiang, Jingxin Qiao, Zeng Wang, Aiping Tong, Jinliang Yang, Shengyong Yang, Li Yang

**Affiliations:** State Key Laboratory of Biotherapy and Cancer Center, West China Hospital, Sichuan University, and Collaborative Innovation Center for Biotherapy, Chengdu, China

**Keywords:** Drug screening, Target identification

**Dear Editor**,

The outbreak of the SARS-CoV-2 epidemic once again demonstrates that RNA viruses, through mutations, genetic recombination and cross-species transmission, can pose a serious threat to the health of people worldwide. Even after the severe acute respiratory syndrome (SARS) and middle east respiratory syndrome (MERS) outbreaks, the world still initially lacked effective means to control the current coronavirus disease 2019 (COVID-19) outbreak. We must work together to develop effective drugs to treat existing and future potential coronavirus infections to reduce their impact on the global health system and human life. Due to time constraints, the ‘conventional drug in new use’ method has become the main method of treating SARS-CoV-2 infections. However, long-term drug development goals should include treatments that can produce broad-spectrum effects on different coronaviruses, and provide the means to alleviate disease symptoms and prevent death.

At present, the potential drug targets of coronavirus are mainly classified into two categories. One target is a viral protease, which controls virus packaging and replication, including 3C-like protease (3CLpro) and papain-like protease (PLpro).^1^ The other is other targets, such as coronavirus spike glycoprotein (S protein), coronavirus receptor (angiotensin-converting enzyme 2, ACE2) and transmembrane protease serine 2 (TMPRSS2), which are closely related to the process of coronaviruses infection of host cells.^2^

In this study, we assessed the anti-coronavirus activity of the antibacterial peptide DP7 (VQWRIRVAVIRK), which was screened and verified by computer simulation in early studies in our laboratory.^3,4^ We studied whether DP7 has the potential to resist coronavirus infections from two aspects, namely, whether it can directly inhibit the activity of coronavirus protease and prevent the binding of SARS-CoV and SARS-CoV-2 S protein to the cell receptor ACE2. Before using DP7 to treat cells, we tested the cytotoxicity of DP7. The results showed that when the DP7 concentration reached 430 μg/ml, 50% of the ACE2-293T cells survived. When the DP7 concentration reached 390.8 μg/ml, 50% of the dendritic cells survived (Fig. 1a and Supplementary Fig. S1). To access whether DP7 has broad-spectrum anti-SARS coronavirus ability, we tested whether DP7 can resist the infection of ACE2-293T by coronavirus S protein pseudovirus and whether DP7 can inhibit cell–cell fusion mediated by coronavirus S protein pseudovirus and ACE2. The results showed that the 50% inhibitory concentration (IC50) of DP7 inhibiting SARS-CoV S protein pseudovirus infected ACE2-293T was 104 μg/ml and that the IC50 of DP7 inhibiting SARS-CoV-2 S protein pseudovirus infected ACE2-293T was 73.625 μg/ml (Fig. 1b, c), and the selectivity index (SI) was 4.13 and 5.8, respectively. However, the control peptide CLS001 did not have a similar effect (Supplementary Fig. S2). Similarly, DP7 has inhibitory activity against SARS-CoV S protein and SARS-CoV-2 S protein-mediated cell–cell fusion with IC_50_ values of 68.38 and 57.6 μg/ml, respectively (Fig. 1d–g and Supplementary Fig. S3a, b), and the selectivity index (SI) was 6.29 and 7.46, respectively. Then, we tested whether DP7 and CLS001 can directly inhibit the binding of SARS-CoV-2 S-RBD and ACE2 through ELISA experiments. The results showed that DP7 can directly inhibit the combination of the two, but CLS001 does not have the ability to inhibit the combination of the two (Supplementary Fig. S4). Subsequently, we further verified the affinity between DP7 and S-receptor-binding domain (RBD)-Fc of SARS-CoV-2 as well as between DP7 and ACE2 through Biacore experiments. The results showed that the affinity of DP7 with S-RBD-Fc of SARS-CoV-2 was 51.9 nM and DP7 with ACE2 was 227 nM (Fig. 1h, i). Next, we performed molecular docking and molecular dynamics (MD) simulations of DP7 and S-RBD of SARS-CoV, S-RBD of SARS-CoV-2 and ACE2 (Fig. 1j–n and Supplementary Tables 1–5). We found that DP7 can act on RBD and ACE2 simultaneously, and the residues W3, R11 and K12 of DP7 interact with RBD as well as ACE2. We also performed docking scoring and binding-free energy calculation of the complex (Supplementary Tables 6 and 8). The binding-free energy of ACE2/DP7 (−115.07 ± 1.68 kcal/mol) and ACE2/SARS-CoV-2 S-RBD (−119.89 ± 2.28 kcal/mol) were not significantly different, indicating that DP7 might potentially inhibit the binding of SARS-CoV S-RBD to ACE2 (Supplementary Table 8). In addition, we also tested whether DP7 can inhibit the activity of SARS-CoV-2 protease by enzyme activity inhibition test. However, although DP7 showed a certain inhibitory activity against SARS-CoV-2-3CLpro (the DP7 concentration that inhibited 50% of the enzyme activity was 66.67 μg/ml), the inhibitory activity did not reach 100% with increasing DP7 concentrations (Fig. 1o, p). In addition, DP7 showed no inhibitory activity against SARS-CoV-2-PLPro (data not shown). Furthermore, we performed MD simulations of SARS-CoV-2-3CLpro and DP7 (Fig. 1q), the binding-free energy was −100.35 ± 1.57 kcal/mol and the docking score was −11.96 kcal/mol (Supplementary Tables 6–8).

**Fig. 1:**
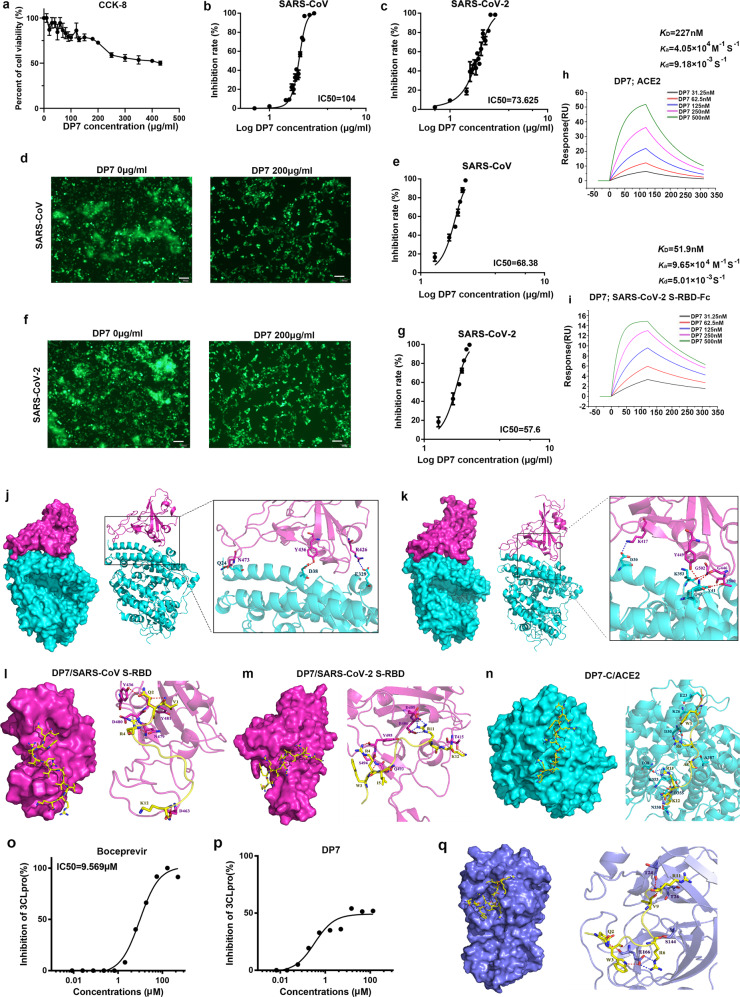
DP7 showed potent inhibitory activity against SARS-CoV and SARS-CoV-2 infection. **a** Cytotoxicity test of DP7. DP7 inhibited the efficiency of (**b**) SARS-CoV S protein pseudovirus and (**c**) SARS-CoV-2 S protein pseudovirus infecting ACE2-293T. DP7 inhibited cell–cell fusion mediated by (**d**, **e**) SARS-CoV S protein and (**f**, **g**) SARS-CoV-2 S protein. Surface plasmon resonance (SPR) sensorgram showing the binding kinetics for DP7 and (**h**) ACE2 and (**i**) S-RBD-Fc of SARS-CoV-2. Binding mode and interactions of ACE2 with (**j**) SARS-CoV S-RBD (PDB ID: 2AJF) and (**k**) SARS-CoV-2 S-RBD (PDB ID: 6LZG). Binding mode and interactions of DP7 with (**l**) SARS-CoV S-RBD, (**m**) SARS-CoV-2 S-RBD and (**n**) ACE2. Inhibitory activity of (**o**) boceprevir and (**p**) DP7 on SARS-CoV-2-3CLpro. **q** The surface binding model of SARS-CoV-2-3CLpro with DP7

We believe that DP7, which has antibacterial activity, exhibits certain advantages in anti-coronavirus infection. In particular, some patients infected with coronavirus may be complicated by co-occurring bacterial infections and should be treated with a combination of antiviral drugs and antibacterial drugs.^5^ In our previous experiments, we confirmed that DP7 was an antibacterial peptide with broad-spectrum antibacterial activity and can resist clinically resistant bacteria. Here, we found that DP7 has the potential to prevent SARS-CoV S protein pseudovirus and SARS-CoV-2 S protein pseudovirus from invading ACE2-293T cells. Therefore, we believe that it may be feasible to use DP7 to combat coronavirus infection in the future.

In conclusion, this study demonstrates the potential effect of DP7 on inhibiting SARS-CoV and SARS-CoV-2 infection from two aspects, i.e., inhibiting SARS-CoV and SARS-CoV-2 S protein pseudovirus infection of ACE2-293T cells, inhibiting SARS-CoV S protein- and SARS-CoV-2 S protein-mediated cell–cell fusion and inhibiting SARS-CoV-2-3CLpro enzyme activity, which provides a theoretical and experimental basis for the further development of DP7 as a drug against coronavirus infections.

## Supplementary information

Supplementary Materials

## Data Availability

The original datasets are available from the corresponding author upon request.
