# Supplementary material for: Antimicrobial peptide DP7 with potential activity against SARS coronavirus infections
Source: Signal Transduct Target Ther. 2021 Apr 1;6:140. doi: 10.1038/s41392-021-00551-1 (PMC8012516; doi:10.1038/s41392-021-00551-1)
Supplement: Supplementary file 1 — Supplementary Materials [file 41392_2021_551_MOESM1_ESM.docx]

Supplementary Materials for

Antimicrobial peptide DP7 with potential activity against SARS coronavirus infections

Rui Zhang^1^, Xiaohua Jiang^1^, Jingxin Qiao^1^, Zeng Wang^1^, Aiping Tong^1^, Jinliang Yang^1^, Shengyong Yang^1^, Li Yang^1^*

^1^State Key Laboratory of Biotherapy and Cancer Center, West China Hospital, Sichuan University, and Collaborative Innovation Center for Biotherapy, Chengdu, 610041, China.

Correspondence to: [Li Yang: yl.tracy73@gmail.com](mailto:Li%20Yang:%20yl.tracy73@gmail.com)

**This PDF file includes:**

Materials and Methods

Figure. S1 to S4

Table S1 to S8

**Materials and methods**

**Cells and culture conditions**

HEK 293T cells stably expressing recombinant human ACE2 (hACE2) (ACE2-293T cells, ACE2 expression was greater than 99%) were established in our lab by overexpression of hACE2[^1^](#_ENREF_1), and we obtained it from Aiping Tong. Dulbecco’s modified Eagle’s medium (DMEM) containing 10% fetal bovine serum (FBS) and 100 units/ml streptomycin and penicillin (PS) was used to culture HEK 293T (American Type Culture Collection, Manassas, VA, USA) and ACE2-293T cells[^1^](#_ENREF_1). All cells were cultured at 37°C in 5% CO_2_. DMEM, FBS and PS were all purchased from Thermo Fisher Scientific.

**Peptides**

DP7 (VQWRIRVAVIRK) and CLS001 (ILRWPWWPWRRK) were synthesized by Shanghai Science Peptide Biological Technology (Shanghai, China) using fluorenylmethyloxycarbonyl chemistry and purified by high-performance liquid chromatography (HPLC) to 99% purity.

**Acquisition and cultivation of bone marrow-derived dendritic cell (BMDC)**

BMDCs were generated from 6- to 8-week-old female mice as previously described[^2^](#_ENREF_2). Briefly, after treating bone marrow cells with red blood cell lysis buffer, approximately 2-3×10^6^ cells were cultured in 10 ml of RPMI 1640 medium containing 20 ng/ml granulocyte-macrophage colony stimulating factor (GM-CSF) (Prime Gene Biotechnology, Shanghai, China), 10% FBS and 1% PS for 8 days to obtain BMDCs.

**Cytotoxicity assay**

The cytotoxicity of DP7 to ACE2-293T cells and mouse bone marrow-derived dendritic cells was tested using Cell Counting Kit-8 (CCK-8; Dojindo, Kumamoto, Japan) according to the instructions. Briefly, 2×10^4^ cells were seeded into the wells of a 96-well microtiter plate and incubated at 37°C for 24h. Then, medium was replaced with DMEM or RPMI 1640 complete medium containing DP7 at graded concentrations. After incubation at 37°C for 24 h, CCK-8 solution (10 μl per well) was added followed by an additional incubation for 1h. The absorbance was measured at 450 nm.

**Plasmids**

The pcDNA3.1 plasmids that encode SARS-CoV S protein (pcDNA3.1-SARS-CoV-S), SARS-CoV-2 S protein (pcDNA3.1-SARS-CoV-2-S) and eGFP (pcDNA3.1-eGFP) were maintained in our laboratory[^1^](#_ENREF_1).

**Inhibition of SARS-CoV and SARS-CoV-2 pseudovirus infection**

The pseudoviruses were produced and titrated using methods similar to that described previously[^1^](#_ENREF_1)^,^[^3^](#_ENREF_3). The SARS-CoV pseudovirus and SARS-CoV-2 pseudovirus were obtained from our laboratory[^1^](#_ENREF_1). Briefly, pcDNA3.1-SARS-CoV-S and pcDNA3.1-SARS-CoV-2-S were transfected into 293T cells using lipofectamine 3000 (Invitrogen) reagents. Then, 24 h after transfection, 293T cells were infected with G*ΔG-VSV with a multiplicity of four. After 2 h, transfected cells were washed with PBS and replaced with fresh culture medium and incubated for 24 h. The culture supernatants containing pseudoviruses were harvested, filtered through 0.45 μM pore-size (Millipore, SLHP033RB) and stored at -80 °C until use. To detect the inhibitory activity of DP7 and CLS001 on SARS-CoV S protein pseudovirus and SARS-CoV-2 S protein pseudovirus infection, ACE2-293T cells were plated at a density of 5×10^4^ cells per well in a 96-well plate for 24 h. Then, the SARS-CoV and SARS-CoV-2 pseudoviruses were mixed with different concentrations of DP7 for 30 min and were transferred to ACE2-293T cells for an additional 72 h. Then, the culture supernatant was aspirated gently followed by adding 30 μl PBS and 30 μl luciferase substrate (Beyotime Biotechnology) in each well. Two min after incubation at room temperature, 50 μl of lysate was transferred to black solid 96-well plates for the detection of luminescence using a microplate luminometer (Promega, Madison, WI, USA). The IC50 was calculated using Graphpad Prism 8.

**Inhibition of SARS-CoV and SARS-CoV-2 S protein-mediated cell-cell fusion**

The inhibitory activity of DP7 on SARS-CoV and SARS-CoV-2 S protein-mediated cell-cell fusion was assessed in this study refer to the previous experimental method[^4^](#_ENREF_4). Briefly, ACE2-293T cells transfected with pcDNA3.1-eGFP served as target cells. To prepare effector cells expressing S protein of coronavirus, 293T cells were transfected with one of the S protein expression vectors, including pcDNA3.1-SARS-CoV-S and pcDNA3.1-SARS- CoV-2-S. For cell-cell fusion assay, effector cells and target cells were co-cultured in DMEM complete medium for 24 h. Then, 293T cells transfected with pcDNA3.1 were used as a negative control. After incubation, five fields were randomly selected in each well to count the number of fused and unfused cells under an inverted fluorescence microscope (Nikon Eclipse Ti-S). The fusion rate was calculated by observing the fused and unfused cells.

**Enzyme-linked Immunosorbent Assay (ELISA)**

The SARS-CoV-2 inhibitor screening kit (Acro, US) and ELISA kit (Solarbio, China) were used to test whether DP7 or CLS001 inhibits the combination of SARS-CoV-2-S protein and ACE2 according to the standard specification. In short, the high binding assay plate was coated with SARS-CoV-2 S protein RBD (0.5 μg/ml), incubated overnight at 4 ℃, and washed thrice with PBST for 5 min each. Next, the blocking liquid was added, and the plate was incubated at 37 ℃ for 1.5 h followed by three washes with PBST for 5 min each. Next, the blocking liquid was added, and the plate was incubated at 37 ℃ for 1.5 h followed by three washes with PBST for 5 min each. Then, DP7 (2 μg/ml) and biotinylated human ACE2 (0.12 μg/ml) were added to the wells, and the plate was incubated at 37 ℃ for 1 h followed by three washes with PBST for 5 min each. Subsequently, streptavidin-HRP (0.1 μg/ml) was added to each well, and the plate was incubated at 37 ℃ in the dark for 1 h followed by three washes with PBST for 5 min each. Next, TMB was added for color development, and termination solution was added to stop color development according to the instructions. Finally, signal reading was performed at 450nm. The SARS-CoV-2 inhibitor (2 μg/ml) (Recombinant protein of ACE2) provided in the kit was used as the positive control.

**Surface plasmon resonance technology-based binding assay and kinetic study**

To detect the interaction of DP7 with ACE2 and SARS-CoV-2 S protein receptor binding domain (RBD; SARS-CoV-2 S-RBD), Biacore^TM^ 8K (GE, USA) based on surface plasmon resonance (SPR) technology was used. SPR analysis was performed at 25°C. ACE2-His (purity≥ 95%; GenStar, China) and SARS-CoV-2 S-RBD-Fc (purity≥ 95%; GenStar, China) were diluted to a final concentration of 20 μg/ml in 10 mM sodium acetate buffer (pH 4.5) and fixed to a CM5 sensor chip (GE, USA) using the standard primary amine coupling method[^5^](#_ENREF_5). PBS-P (0.02 M phosphate, 0.137 M NaCl, 27 mM KCl, pH 7.4, and 0.05% P20) was used as the running buffer. DP7 was diluted to 31.25, 62.5, 125, 250, and 500 nM with running buffer. Different DP7 concentrations flowed over the CM5 sensor chip, and the flow rate was set as 30 μl/min. The flow time was set as 120 s, and the dissociation time was set as 180 s. Then, DP7 samples flowed over the sensor chip in 10 mM glycine-HCl (pH 1.7). The flow rate was set as 30 μl/min, and the flow time was 30 s. Finally, the response value was obtained, and a 1:1 binding model from the Bia-evaluation analysis software was used to calculate the dynamic parameters and compare the affinity of DP7.

### Molecular docking

### MOE-Dock was used for protein-peptide docking of the DP7 peptide using the three proteins and for protein-protein docking of the SARS-CoV-2 S protein with ACE2. This information was used to predict the initial binding positions for further MD simulations.

For protein-peptide docking, the three-dimensional (3D) structure of DP7 was built in MOE through the protein build module and energy minimization calculation. The X-ray structure of SARS-CoV-2-3CLpro was downloaded from RCSB Protein Data Bank (PDB ID: 6LU7), and the X-ray structures of SARS-CoV S-RBD and SARS-CoV-2 S-RBD were also utilized (PDB IDs: 2AJF and 6LZG, respectively). Then, the protonation state of the target proteins and the orientation of the hydrogens were optimized by LigX at a pH of 7 and temperature of 300 K. The binding site of SARS-CoV-2-3CLpro was defined around the position of the ligand in the original X-ray structure. The binding sites of SARS-CoV S-RBD and SARS-CoV-2 S-RBD were defined around the position of residues involved in the binding of the protein-protein interaction. Prior to docking, the force field of AMBER10: EHT and the implicit solvation model of the reaction field (R-field) were selected. The docking workflow followed the “induced fit” protocol, in which the side chains of the receptor pocket were allowed to move according to ligand conformations with a constraint on their positions. The weight used for tethering side chain atoms to their original positions was 10. All docked poses of molecules were first ranked by the London dG scoring function, and force field refinement was performed on the top 30 poses followed by a rescoring by the GBVI/WSA dG scoring function. Then, the best-ranked pose was finally selected as the binding pose for further MD simulations. For protein-protein docking, the protein ACE2 was defined as the target, and SARS-CoV S-RBD and SARS-CoV-2 S-RBD were defined as ligands. The binding site and final binding position were selected with reference to the X-ray structure of ACE2 binding with SARS-CoV S-RBD (PDB ID: 2AJF) and SARS-CoV-2 S-RBD (PDB ID: 6LZG). The final binding position was further optimized by MD simulations.

### MD simulations

The above protein-peptide and protein-protein complexes were optimized by MD simulations. Each of the complexes was neutralized by adding sodium/chlorine counter ions and solvated in a cuboid box of TIP3P water molecules with 10 Å solvent layers between the box edges and solute surface. All MD simulations were performed using AMBER16[^6^](#_ENREF_6). The AMBER FF14SB force field was applied, and the SHAKE algorithm was used to restrict all covalent bonds involving hydrogen atoms with a time step of 2 fs. The particle mesh Ewald (PME) method was employed to treat long-range electrostatic interactions. For each solvated system, two minimization steps were performed before the heating step. The first 4,000 cycles of minimization were performed with all heavy atoms restrained with 50 kcal/(mol·Å2), whereas solvent molecules and hydrogen atoms were free to move. Then, non-restrained minimization was performed involving 2,000 cycles of steepest descent minimization and 2,000 cycles of conjugated gradient minimization. Afterwards, the whole system was first heated from 0 K to 300 K in 50 ps using Langevin dynamics at a constant volume and then equilibrated for 400 ps at a constant pressure of 1 atm. A weak constraint of 10 kcal/(mol·Å2) was used to restrain all the heavy atoms during the heating steps. Periodic boundary dynamics simulations were performed for the whole system with an NPT ensemble (constant composition, pressure, and temperature) at a constant pressure of 1 atm and constant temperature of 300 K in the production step. In the production phase, a 100 ns simulation was performed. The binding free energy of the complex was calculated using the MM-PBSA method.

**SARS-CoV-2-3CLpro activity assay**

SARS-CoV-2-3CLpro (purity ≥ 95%; specific activity >13,000 pmol/min/mg) was obtained from Novoprotein (Shanghai, China). The fluorogenic substrate Dabcyl-KNSTLQSGLRKE- Edans was used to measure the enzymatic activity of SARS-CoV-2-3CLpro by cleavage based on previously reported protocols[^7-9^](#_ENREF_7). Briefly, the inhibition of SARS-CoV-2-3CLpro activity was assayed by preincubating the enzyme (0.4 µM) with different concentrations of DP7 and boceprevir (a positive control) (MCE, USA) at room temperature for 10 min. Then, 10 µM of fluorogenic substrate (Dabcyl-KNSTLQSGLRKE- Edans) was added to initiate the reaction, and the change in the fluorescence intensity was monitored on a GENios microplate reader (Tecan, Mannedorf, Switzerland). Enzyme activity was determined based on the increase in fluorescence upon continuous monitoring of the reactions in 96-well black microplates (BMG LABTECH, Offenburg, Germany) using wavelengths of 340 nm and 490 nm for excitation and emission, respectively. Finally, Origin 7.0 software was used to calculate the concentration of inhibitor producing 50% inhibition.

**Statistical analysis**

All data were evaluated and plotted using GraphPad Prism 6.0. Student's t-test and two-way ANOVA were used to analyze differences in data. A *p-*value < 0.05 was considered statistically significant.


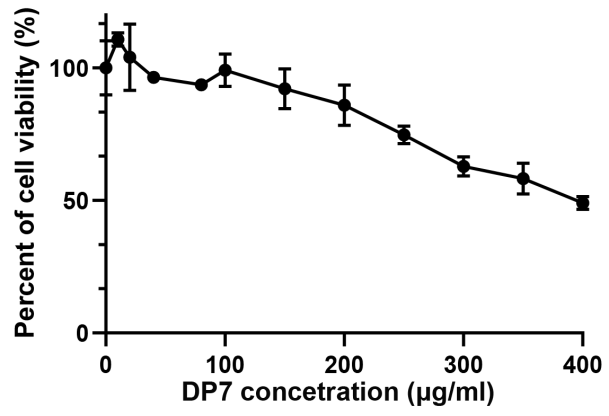


**Figure. S1**

Cytotoxicity test of DP7 using bone marrow-derived dendritic cells.


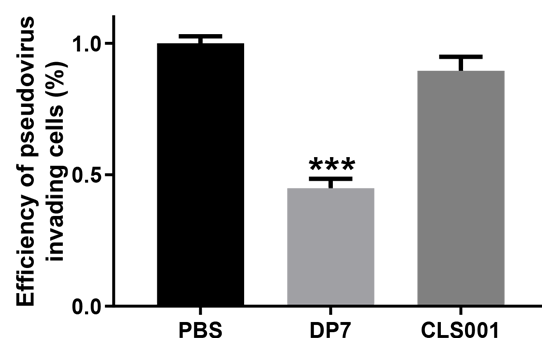


**Figure. S2**

The efficiency of DP7 (73.625 μg/ml) and CLS001 (120 μg/ml) in inhibiting SARS-CoV-2 pseudovirus infection.


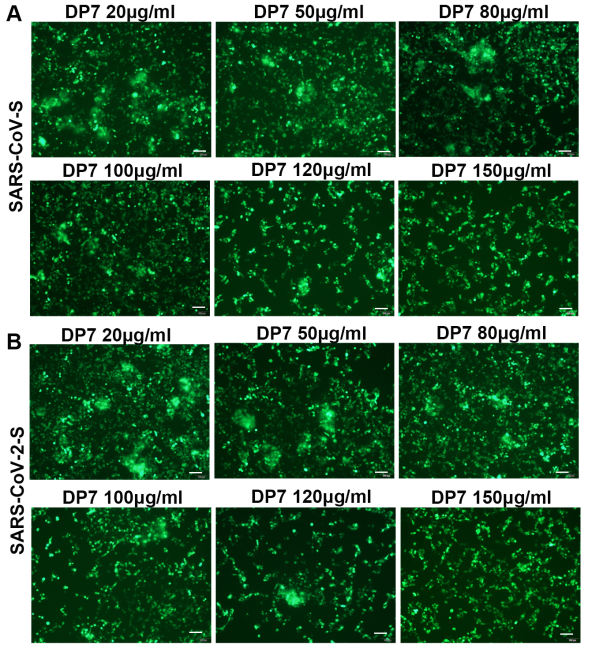


**Figure. S3**

DP7 inhibited cell-cell fusion mediated by (A) SARS-CoV S protein and (B) SARS-CoV-2 S protein.


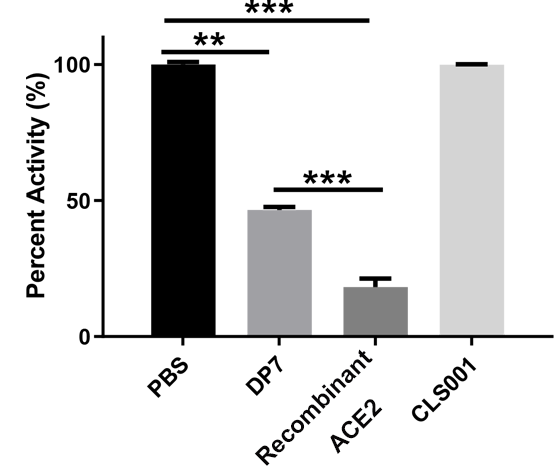


**Figure. S4**

Detection of the DP7-mediated inhibition of SARS-CoV-2 S-RBD binding to ACE2 by ELISA.

**Table S1.** The contact list between ACE2 and SARS-CoV-S-RBD

| Chain 1 | Residue | Chain 2 | Residue | Interaction type |
| --- | --- | --- | --- | --- |
| ACE2 | Asp38.OD2 | S-RBD | Tyr436.OH | Hydrogen bond interaction |
| ACE2 | Gln42.OE1 | S-RBD | Tyr436.OH | Hydrogen bond interaction |
| ACE2 | Gln325.OE1 | S-RBD | Arg426.NH2 | Hydrogen bond interaction |
| ACE2 | Glu329.OE1/OE2 | S-RBD | Arg426.NH2 | Salt bridge |

**Table S2.** The contact list between ACE2 and SARS-CoV-2-S-RBD.

| Chain 1 | Residue | Chain 2 | Residue | Interaction type |
| --- | --- | --- | --- | --- |
| ACE2 | Tyr41.OH | SARS-CoV-2-S-RBD | Thr500.OG1 | Hydrogen bond interaction |
| ACE2 | Gln42.NE2 | SARS-CoV-2-S | Gly446.O | Hydrogen bond interaction |
| ACE2 | Gln42.NE2 | SARS-CoV-2-S | Tyr449.OH | Hydrogen bond interaction |
| ACE2 | Lys353.O | SARS-CoV-2-S | Gly502.N | Hydrogen bond interaction |
| ACE2 | Asp30.OD1 | SARS-CoV-2-S | Lys417.NZ | Salt bridge |

**Table S3.** The contact list between DP7 and SARS-CoV-S-RBD

| Chain 1 | Residue | Chain 2 | Residue | Interaction type |
| --- | --- | --- | --- | --- |
| S-RBD | Tyr481.O | DP7 | Val1.N | Hydrogen bond interaction |
| S-RBD | Tyr436.OH | DP7 | Gln2.OE1 | Hydrogen bond interaction |
| S-RBD | Asn479.OD1 | DP7 | Arg4.NE | Hydrogen bond interaction |
| S-RBD | Asp463.OD2 | DP7 | Lys12.N | Hydrogen bond interaction |
| S-RBD | Asp480.OD2 | DP7 | Arg4.NH1 | Salt bridge |

**Table S4.** The contact list between SARS-CoV-2-S-RBD and DP7.

| Chain 1 | Residue | Chain 2 | Residue | Interaction type |
| --- | --- | --- | --- | --- |
| SARS-CoV-2-S-RBD | Thr415.OG1 | DP7 | Lys12.NZ | Hydrogen bond interaction |
| SARS-CoV-2-S-RBD | Gln493.NE2 | DP7 | Ile5.O | Hydrogen bond interaction |
| SARS-CoV-2-S-RBD | Ser494.N | DP7 | Trp3.O | Hydrogen bond interaction |
| SARS-CoV-2-S-RBD | Tyr495.O | DP7 | Arg4.NH1 | Hydrogen bond interaction |
| SARS-CoV-2-S-RBD | Asp405.OD2 | DP7 | Arg11.NH1/NH2 | Salt bridge |
| SARS-CoV-2-S-RBD | Glu406.OE1/OE2 | DP7 | Arg11.NH2/NE | Salt bridge |

**Table S5.** The contact list between ACE2 and DP7.

| Chain 1 | Residue | Chain 2 | Residue | Interaction type |
| --- | --- | --- | --- | --- |
| ACE2 | Glu23.OE1 | DP7 | Trp3.N | Hydrogen bond interaction |
| ACE2 | Lys26.NZ | DP7 | Trp3,O | Hydrogen bond interaction |
| ACE2 | Asp30.OD2 | DP7 | Trp3.NE1 | Hydrogen bond interaction |
| ACE2 | Asn330.OD1 | DP7 | Lys12.NZ | Hydrogen bond interaction |
| ACE2 | Lys353.O | DP7 | Arg11.N | Hydrogen bond interaction |
| ACE2 | Ala387.O | DP7 | Ala8.N | Hydrogen bond interaction |
| ACE2 | Asp30.OD1/OD2 | DP7 | Arg6.NH2/NE | Salt bridge |
| ACE2 | Asp38.OD1 | DP7 | Arg11.NH1/NH2 | Salt bridge |
| ACE2 | Asp355.OD1/OD2 | DP7 | Lys12.NZ | Salt bridge |

**Table S6.** Docking scores of DP7 and protein.

| Ligands | Receptors | Docking score (kcal/mol) |
| --- | --- | --- |
| DP7 | ACE2 | -11.62 |
| DP7 | SARS-CoV-S-RBD | -10.29 |
| DP7 | SARS-CoV-2-S-RBD | -10.43 |
| DP7 | SARS-CoV-2-3CLpro | -11.96 |

**Table S7.** The contact list between SARS-CoV-2-3CLpro and DP7.

| Chain 1 | Residue | Chain 2 | Residue | Interaction type |
| --- | --- | --- | --- | --- |
| 3CLpro | Thr24.O | DP7 | Arg11.N | Hydrogen bond interaction |
| 3CLpro | Thr26.N | DP7 | Val9.O | Hydrogen bond interaction |
| 3CLpro | Ser144.OG | DP7 | Arg6.O | Hydrogen bond interaction |
| 3CLpro | Glu166.O | DP7 | Gln2.NE2 | Hydrogen bond interaction |
| 3CLpro | Glu166.OE2 | DP7 | Trp3.NE1 | Hydrogen bond interaction |
| 3CLpro | Glu166.OE1/OE2 | DP7 | Arg6.NH2/NE | Salt bridge |

**Table S8**. Average binding energy and its components obtained from the MM-PBSA calculation for the complexes.

| Contribution | Energy (kcal/mol) | | | |
| --- | --- | --- | --- | --- |
|  | ACE2-DP7 | SARS-CoV-2-3CLpro-DP7 | SARS-CoV-2-S-RBD -DP7 | SARS-CoV-2-S-RBD  -ACE2 |
| ∆E_vdw_ | -75.71±0.64 | -81.07±0.66 | -60.75±1.12 | -91.17±1.19 |
| ∆E_elec_ | -1109.65±3.96 | -319.97±3.85 | -227.84±4.42 | -493.84±6.42 |
| ∆G_polar_ | 1127.74±3.44 | 361.12±3.43 | 261.22±4.59 | 530.95±5.85 |
| ∆G_nonpolar_ | -57.46±0.33 | -60.43±0.36 | -54.89±0.80 | -65.83±0.71 |
| ∆G_total_ | -115.07±1.68 | -100.35±1.57 | -82.27±1.91 | -119.89±2.28 |

**References**

1 Yang, J. *et al.* A vaccine targeting the RBD of the S protein of SARS-CoV-2 induces protective immunity. *Nature*. **586**, 572-577, (2020).

2 Zhang, R. *et al.* Cholesterol-modified DP7 enhances the effect of individualized cancer immunotherapy based on neoantigens. *Biomaterials*. **241**, (2020).

3 Nie, J. H. *et al.* Establishment and validation of a pseudovirus neutralization assay for SARS-CoV-2. *Emerg Microbes Infec*. **9**, 680-686, (2020).

4 Xia, S. *et al.* Inhibition of SARS-CoV-2 (previously 2019-nCoV) infection by a highly potent pan-coronavirus fusion inhibitor targeting its spike protein that harbors a high capacity to mediate membrane fusion. *Cell Res*. **30**, 343-355, (2020).

5 Johnsson, B., Lofas, S. & Lindquist, G. Immobilization of proteins to a carboxymethyldextran-modified gold surface for biospecific interaction analysis in surface plasmon resonance sensors. *Analytical biochemistry*. **198**, 268-277, (1991).

6 Case, D. A. *et al.* The Amber biomolecular simulation programs. *Journal of computational chemistry*. **26**, 1668-1688, (2005).

7 Chen, L. L. *et al.* Cinanserin is an inhibitor of the 3C-like proteinase of severe acute respiratory syndrome coronavirus and strongly reduces virus replication in vitro. *J Virol*. **79**, 7095-7103, (2005).

8 Chen, S. *et al.* Enzymatic activity characterization of SARS coronavirus 3C-like protease by fluorescence resonance energy transfer technique. *Acta Pharmacol Sin*. **26**, 99-106, (2005).

9 Chen, L. L. *et al.* Discovering severe acute respiratory syndrome coronavirus 3CL protease inhibitors: Virtual screening, surface plasmon resonance, and fluorescence resonance energy transfer assays. *J Biomol Screen*. **11**, 915-921, (2006).
